# Diseases of the Skin

**Published:** 1888-06

**Authors:** 


*Diseases of the Skin.* By W. ALLAN JAMIESON, M.D., F.R.C.P. Ed. Edinburgh: YOUNG J. PENTLAND. 1888.

Dermatologists rarely get such an *édition de luxe* as this volume, which will still further enhance the reputation of its publisher. The book is charmingly printed. Its essentially practical character will do much to drive away the thought that will come into the minds of many—that surely another general book on skin-diseases was not wanted. Enough etiology and pathology are given without being a burden to the enquirer, and the latest therapeutics of the skin are well recorded. Taking into consideration the marked idiosyncrasies of skin-diseases and their possessors, perhaps there is scarcely enough detail of alternative treatment to satisfy a bewildered doctor, who finds ordinary modes of attack unsuccessful. This is more particularly the case in reference to parasitical diseases. The coloured illustrations show much care in their preparation, and have the advantage of being less unlike the actual life-appearances than most of such productions. If the practitioner's library is to contain only one book on diseases of the skin, it would be well furnished with this book. It does not slavishly follow recognised authorities, is far from being a compilation from other writers, and is largely based on the author's personal experience at Edinburgh.

